# Pairwise covariance adds little to secondary structure prediction but improves the prediction of non-canonical local structure

**DOI:** 10.1186/1471-2105-9-429

**Published:** 2008-10-10

**Authors:** Christopher Bystroff, Bobbie-Jo Webb-Robertson

**Affiliations:** 1 Departments of Biology and Computer Science, Center for Biotechnology and Interdisciplinary Studies, Rensselaer Polytechnic Institute, Troy NY, USA; 2 Computational Biology and Bioinformatics, Pacific Northwest National Laboratory, Richland, WA, USA

## Abstract

**Background:**

Amino acid sequence probability distributions, or profiles, have been used successfully to predict secondary structure and local structure in proteins. Profile models assume the statistical independence of each position in the sequence, but the energetics of protein folding is better captured in a scoring function that is based on pairwise interactions, like a force field.

**Results:**

I-sites motifs are short sequence/structure motifs that populate the protein structure database due to energy-driven convergent evolution. Here we show that a pairwise covariant sequence model does not predict alpha helix or beta strand significantly better overall than a profile-based model, but it does improve the prediction of certain loop motifs. The finding is best explained by considering secondary structure profiles as multivariant, all-or-none models, which subsume covariant models. Pairwise covariance is nonetheless present and energetically rational. Examples of negative design are present, where the covariances disfavor non-native structures.

**Conclusion:**

Measured pairwise covariances are shown to be statistically robust in cross-validation tests, as long as the amino acid alphabet is reduced to nine classes. An updated I-sites local structure motif library that provides sequence covariance information for all types of local structure in globular proteins and a web server for local structure prediction are available at .

## Background

A key challenge of the post genomic era is the prediction of protein structure. Since X-ray crystallography and NMR-based methods are still relatively low throughput, computational inference approaches are of the upmost importance. One of the key approaches to this problem has been to describe fragments that represent specific local structural elements in libraries [1,2]. Fragments can be used as input to folding simulation algorithms such as Rosetta, TASSER and SimFold [3-6].

I-sites motifs represent small, independently folding substructures in proteins and may play a role in initiating the folding process[2]. The short amino acid sequence patterns associated with I-sites motifs have been found to correlate with common local structures in proteins and match short local structures, such as helix caps and beta-turns, with sequence probability distributions, or *profiles*. The profiles can be used to predict the local structure given a sequence. I-sites motifs were found by clustering short sequence patterns from proteins of known structure after factoring out redundancy due to homologous relationships. Because the patterns are recurrent, and yet clustered from non-homologous proteins, the motifs are almost certainly the results of convergent, not divergent, evolution. Local energetic preferences are most likely at the root of the selective pressure leading to the convergent evolution.

I-sites motifs represent small, independently folding substructures in proteins and may play a role in initiating the folding process. In molecular dynamics simulations, peptide sequences with higher I-sites scores correlated with a greater equilibrium stability [7]. In experimental studies, peptides with I-sites motif patterns were found to fold to their corresponding motif structures in isolation[8]. Blind predictions of local structure were found to have approximately 44% accuracy when measured as the fraction of the sequence that falls within correct 8-residue segments [9]. Randomly selected 8-residue segments have an average 5.7% prediction accuracy.

The scoring function for I-sites predictions may be viewed as a knowledge-based energy function, since a high score of a sequence to an I-sites motif implies a high probability that the sequence segment adopts the motif local structure. A high probability implies a low free energy as an autonomous folding unit. If the I-sites scoring function is capturing an energetic quantity, then might we do better by re-structuring the scoring function to resemble a force field?

A force field generally functions on pairs of atoms rather than on single atoms. To better resemble an energy calculation, the I-sites scoring function should act on pairs of amino acid residues rather than on single residues. A covariant statistical interaction should imply an energetic interaction. That is, amino acids that occur together more often than expected by chance imply the existence of an attractive force, while pairs that occur less often than expected imply a repulsive force.

If energetic stability was the selective pressure for the covergent evolution of local structure motifs, then we can view these motifs as minima in a broad sequence/structure energy landscape. Evolving sequences preferentially populated the energy valleys over time. If the sampling of this landscape is representative, then the statistical probabilities (*p*) are convertible to energies using the Gibbs equation, ΔG = -RTlog(*p*/1-*p*). The number of occurrences of any pair of amino acids in the context of a structure should be a good measure of their physical interaction energy. Hydrophobic contacts, salt bridges and other energetic interactions that contribute to the stability of a local structure should be over-represented in the statistics, while destabilizing interactions such as like-charges should be under-represented. Deriving an energy-like score from pairwise sequence statistics is quite different from independently summing probabilities from single positions and calculating a log-likelihood score. The latter approach tacitly and erroneously implies that the hydrophobic effect decreases only by half when one of the pair of interacting hydrophobic sidechains is removed, instead of decreasing to zero, as we know it does.

Positional pairwise sequence covariance has been shown to be a weak predictor of residue-residue tertiary contacts within protein families in several studies [10,11], probably because such paired mutations are rare in evolutionary history. In the present work, however, no mutational pathway is implied since the observed sequences are not the results of divergent evolution. Each example in the training set comes from a different ancestral line.

In this work, the I-sites motif library has been modified and refined by adding a pairwise covariance metric to each motif. Covariances are expressed in the model as a four-dimensional tensor in which each element is a pairwise positional correlation between two amino acid profile classes. The scoring matrices were trained using expectation/maximization to predict the backbone dihedral angles in a non-redundant set of proteins. The results on an independent test set are reported for three different training strategies.

## Results and Discussion

Significant improvements in prediction over the previous I-sites method were obtained by simply using a better model for measuring confidence and by retraining the I-sites profiles on a larger and newer dataset (ISL5.1). An overall improvement was found when covariance was added to the profile model without re-training the profiles (ISL5.3). Adding covariance tensors and re-training the profiles (ISL5.2) improved prediction accuracy the most, but only slightly more than by retraining the profiles alone. Interestingly, the contribution of covariance to prediction accuracy was found to be positive and significant for non-periodic local structure motifs, whereas prediction accuracy of canonical secondary structure elements (helix and sheet) was not greatly improved by covariance, even though each of the individual motifs showed improved prediction after adding covariance. Over-fitting was present in some cases, but does not fully explain the shortfall in prediction, which was observed in both training and test data.

Several trends were observed in nearly all motifs. The sign of pairwise correlation and the pair of amino acid classes involved usually made good chemical sense. For example, we consistently observed a positive correlation value for hydrophobic sidechains that were in contact in alpha helices.

Table 1 shows the reduced alphabet of nine amino acid profile classes used for calculating covariance. Reducing the alphabet size from 20 to 9 was critical for statistical significance in this study. The number of classes for this study (9) was selected empirically. In preliminary studies, an alphabet size of 20 and 8 both produced inferior test results on selected motifs. [For details on how the classes were defined see Additional File 1]

**Table 1:** Area under ROC curve

	**Training Set**	**Test Set**
**ISL 3.1**	0.696	0.709
**ISL 5.1**	0.730	0.735
**ISL 5.2**	0.732	0.728
**ISL 5.3**	0.719	0.718

### Improvements in local structure prediction

**Figure 2:**
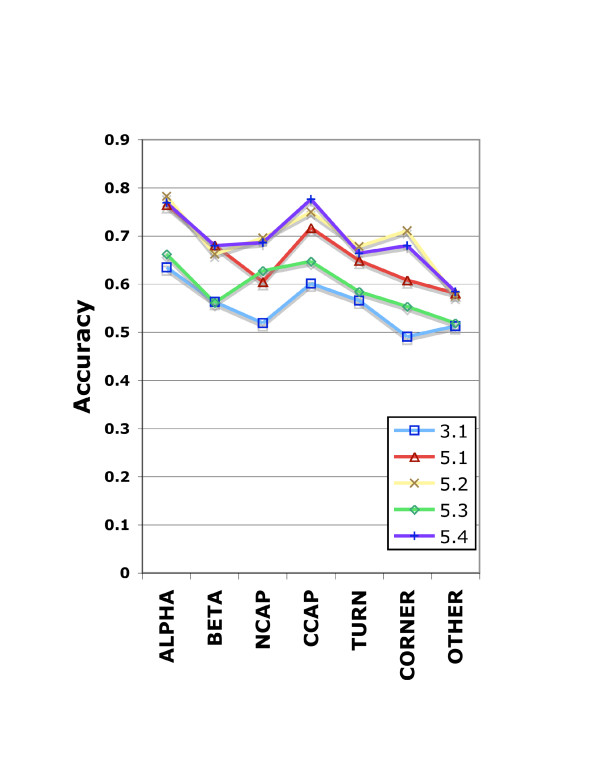
**Accuracy versus motif type versus I-sites library training strategy**. Numbers indicate the library version. ALPHA: motifs having all helical angles. BETA: motifs having all beta sheet angles. NCAP: Loop motifs at the N-terminus of helices. CCAP: Loop motifs at the C-terminus of helices. TURN: motif.

Motif prediction accuracy was evaluated for the original and the three new libraries trained. The correctness of a motif prediction was defined using the maximum backbone angle deviation (MDA) metric. But motif predictions may overlap, leading to ambiguity. To assure that each position was counted only once, we assigned each position the value (true or false) of the highest confidence overlapping motif prediction. These predictions were collected for the entire dataset and sorted by confidence. The overall accuracy was plotted for each of the broad classes of local structure (Figure 2) for each of the four libraries.

**Figure 3:**
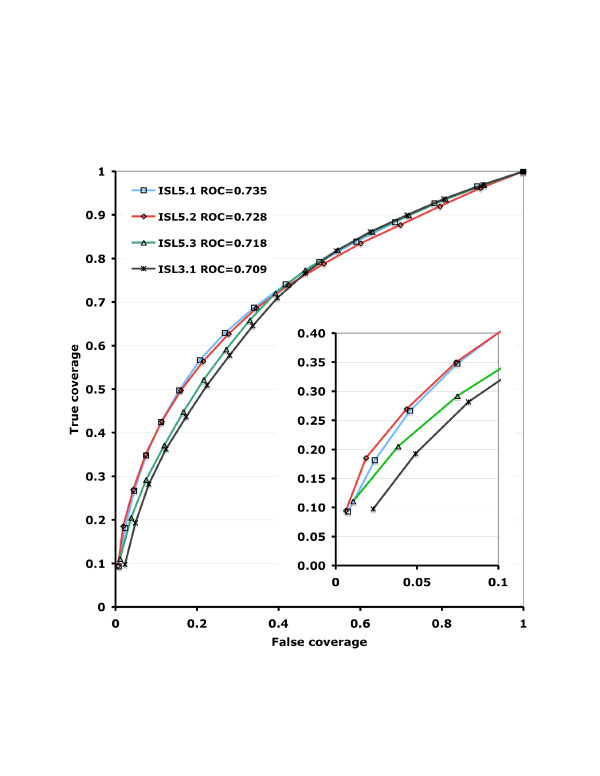
**True versus false coverage for all libraries**. Overall accuracy is measured as the area under the ROC curve. Inset: Blowup of high confidence region.

Additionally, we evaluated *confidence *as a classifier of true and false predictions using a Receiver Operating Characteristic (ROC) curve [12]. Figure 3 summarizes the ROC results over all I-sites motifs for the previously described library, version 3.1, and for each of the new I-sites libraries, ISL 5.1, ISL 5.2 and ISL 5.3, which were trained in different ways. The new motif libraries have a higher overall accuracy and have a greater fraction of high confidence predictions. ISL 5.1 had the highest overall ROC (0.735), followed by ISL 5.2 (0.728), ISL 5.3 (0.718) and the starting library ISL 3.1 (0.709). But although the library with the covariance model was slightly worse overall as a predictor of local structure, it did significantly better at predicting loop and cap motifs than ISL 5.1. The improvements were outnumbered, however, by poorer predictions of beta strands by ISL 5.2. The accuracy of the highest confidence predictions was higher for ISL 5.2 than for ISL 5.1 or any of the other libraries, as seen in the inset in Figure 3.

ROC and accuracy were measured on test set data, a set of proteins not used in training. To assess the potential over-fitting of the data, we compared prediction accuracy on the training and test set data (Table 1). Differences between these numbers in the libraries containing covariance appear to indicate some over-fitting, but only slight. The overall improvements in accuracy are seen in the test data, showing that the improvements are not the result of over-fitting.

#### Case study: Type-I' beta-hairpin motif

**Figure 1:**
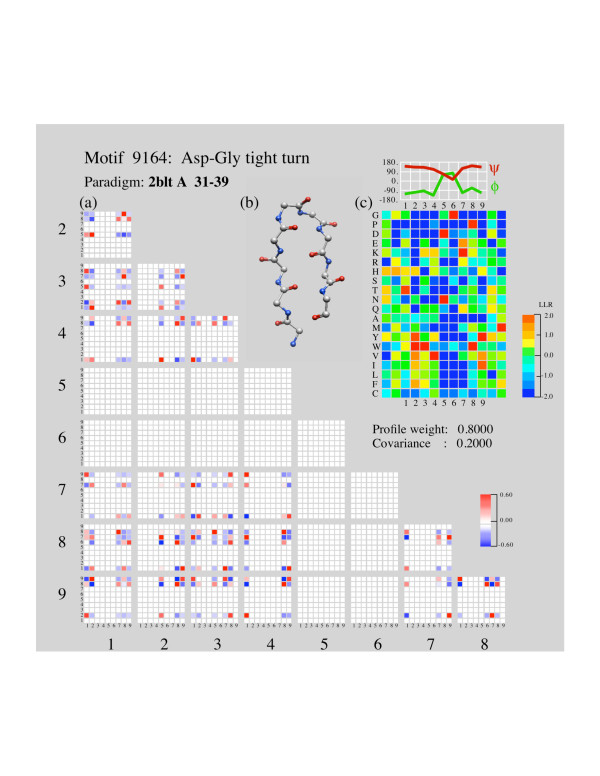
**Type-I' beta hairpin motif**. Type-I' beta hairpin (DG hairpin) from ISL5.2. (a) Correlation tensor. Large numbers indicate positions in the motif, small numbers indicate amino acid class. Correlation is expressed as a color from blue to red according to the scale in the lower right. (b) ball-and-stick rendering of the motif structure. (c) Profile expressed as log-likelihood ratios according to the color scale at the right. Amino acids are arranged roughly from non-polar on the bottom to polar on the top. Plotted above are backbone angles for each position.

Although it is not possible to discuss all of the local structure motifs, we can see in a case study that some features of covariant sequence patterns agree with chemical intuition. As an example, consider the type-I' beta hairpin motif. This motif carries a strong preference for the two-character sequence [DN]G in positions 2 and 3 of the turn, where consecutive backbone phi angles are positive. Since the sequences do not vary in these positions, they also do not co-vary, and therefore no correlations were observed for those positions (Figure 1). But in the other motif positions, positive correlations occur that would favor having the sidechains together on the same side of the hairpin, and negative correlations occur that would favor contacts where the motif does not have contacts.

Positions 3 and 8 have sidechains that fall on the same side of the 2-stranded sheet and are in contact, and a positive correlation is found between a positive charge (class 1) at position 3 and a negative charge (class 7) at position 8, – a salt bridge. The reversed charge placements are also favored, but like charges at these positions are disfavored.

Positions 7 and 8 point in opposite directions. Here like charges are positively correlated and opposite charges are negatively correlated. Opposite charges would attract, possibly putting positions 7–8 into a turn or bulge conformation instead of a strand. This is an example of negative design. [For a second case study see Additional File 1]

### Comparison of three libraries

The starting motif library, ISL 3.1 was reported in 2000 along with HMMSTR, a hidden Markov model for local structure that is ILS 4 [13], but was not assessed here because it carries additional information about motif adjacencies. The new libraries were trained using an expectation/maximization approach, using three different strategies, which had different effects on the overall accuracy across local structures.

#### ISL 5.1: Profile refined only

As a control experiment in this attempt to demonstrate sequence covariance in local structure, the previous library was updated using only the profile information. The results show that updating the library greatly improved the overall prediction performance. Over the years since I-sites was originally trained, the size of the PDBselect25 database has grown by about 3-fold. Updating the profiles by iterative retraining led to improved accuracy across all local structure types as seen in Figure 2.

#### ISL 5.2: Tensor and profile refined together

The second training strategy was to update both the correlation tensor and the amino acid profile at each iteration. We observed that the profiles in library 5.2 were often lower in information content (higher entropy) than the profiles in the libraries 5.1 and 5.3 showing that pairwise correlations can replace the information in a profile and are often sufficient to predict the structure of a local motif. Motifs containing conserved glycines or prolines held more of the scoring function weight (Eq. 10) in the profile score than motifs that did not contain a glycine or proline. Note that, since they do not vary, they cannot co-vary.

### ISL 5.3: Tensor refined, profile fixed

ISL 5.3, in which the tensor was iteratively updated but the profile was not, was found to have a lower prediction performance than both Libraries 5.1 and 5.2. By doing this second control experiment, we can see that the improvements in prediction accuracy for helix and strand motifs in version 5.2 are accounted for by the refinement of the profiles, as in library 5.1, not by the addition of the covariance tensor. However, we see improvements in accuracy in the prediction of most loop motifs, including helix caps. A possible rationale for this result is that loop motifs are not repeating structures, while beta strands and alpha helices do contain a repeating sequence pattern. A profile, viewed as an all-or-none model, is a better predictor of a multiposition pattern.

## Conclusion

Overall we observe that there is little benefit in using pairwise correlation to model the local structural motifs in the I-sites library (I-sites 2008). Furthermore, we conclude from this experiment that, in canonical secondary structure, the profile and the correlation tensor are both being fit to an underlying multivariant sequence-structure relationship, which is better captured by a library of profiles of than by a profile plus a correlation tensor.

To understand this view, consider that both canonical secondary structures, helix and strand, are repeating patterns. A register shift of one residue down the chain produces a new pattern that is changed in all positions, not just two. I-sites already contains multiple solutions for helix and strand, each a different length or register shift. A library of profiles is, in essence, a multivariant model composed of discrete, all-or-none solutions. Although we saw significant covariances within each of the helix/strand motifs and each individual motif improved in its performance, the additional peripheral instances that were captured by the improved motif model were already captured by one of the other helix/strand motif profiles in the library. Thus improving the performance of each motif did not improve the preformance overall.

For non-canonical local structure motifs, the story is different. These motifs are not repeat patterns, and therefore one instance cannot match multiple register shifts of one motif, only one. By adding covariance to the model, we were able to capture sequence variants that were not already captured by another motif in the library, and performance on these structures improved.

The results are consistent with protein local structures having arisen by convergent evolution, not by duplication followed by uncorrelated or correlated mutations. The variations in sequence space for each type of local structure (motif) are best modeled by multiple independent sequence profiles, and less well modeled by a single profile with a pairwise covariance tensor.

## Methods

### Amino acid profiles

The probability of an amino acid at a position in the sequence was calculated from the Psi-Blast multiple sequence alignment as the sum of the sequence weights over all sequences having that amino acid, or

(1) $$P_{ia}=\sum_{\begin{array}{l} k\in aligned\\ sequences,s \end{array}}\left\{\begin{array}{cc} if(s_{ik}=a) & w_{k}\\ else & 0 \end{array}\right\}/\sum_{\begin{array}{l} k\in aligned\\ sequences,s \end{array}}\left\{\begin{array}{cc} if(s_{ik}\neq gap) & w_{k}\\ else & 0 \end{array}\right\}$$

where *a *is an amino acid and *s*_*ik *_is a *i*^th ^character in the *k*^th ^sequence. Position-specific sequence weights, *w*_k_, were calculated using the normalized sum of pairwise mismatch distances [14].

Motif profiles were calculated from aligned sequence segments in a similar way, except that equal weighting was applied to each instance of the motif. Thus the motif profile was simply the average profile of the instances of the motif from the training set. One instance of a motif consists of *L*+4 consecutive profile positions, where *L *is the length of the motif structure. Two-residue extensions N and C terminal to the motif structure helped to improve local structure prediction in the previous study [13] and this strategy was retained in this study. Covariances in these terminal positions were ignored.

The amino acid profile for a motif *m *is defined as

(2) $$P_{ja}^{m}=\frac{1}{N_{m}}\sum_{\begin{array}{l} k\in \text{instances}\\ \text{of motif }m \end{array}}P_{ja}^{\text{k}}$$

where *j *is a position from -2 to *L*+1 relative to the start of the motif structure for instance *k *of motif *m*. The background frequencies *F *for each amino acid were averaged over the entire database.

(3) $$F_{a}=\frac{1}{N}\sum_{\begin{array}{l} k\in all\\ positions \end{array}}P_{ka}$$

### Defining amino acid profile classes

K-means clustering was used to partition the space of all single-position profiles, using the similarity score *S*.

(4) $$S(i,j)=\sum_{a=1,20}\log\left(\frac{P_{ia}+\alpha F_{a}}{(1+\alpha )F_{a}}\right)\log\left(\frac{P_{ja}+\alpha F_{a}}{(1+\alpha )F_{a}}\right)$$

where *i *and *j *are any two positions in the training set and *α *= 0.5. This is the same scoring function used to generate the original I-sites clusters [2]. In the K-means algorithm K = 9 was used, based on intuitive knowledge of the diversity of amino acid sidechain chemistries. In the resulting nine clusters, no two clusters shared the same chemical nature, and no single cluster, except one, contained amino acids of very different chemical nature. Table 2 shows the profiles for the 9 amino acid classes.

**Table 2:** Amino acid profile classes

**Class**	**Sidechain characteristics**	**Abundance**	**A**	**C**	**D**	**E**	**F**	**G**	**H**	**I**	**K**	**L**	**M**	**N**	**P**	**Q**	**R**	**S**	**T**	**V**	**W**	**Y**
1	Pos. charge	0.1710	.04	.00	.01	.05	.01	.01	.01	.01	**.26**	.03	.01	.01	.01	**.15**	**.29**	.02	.02	.02	.00	.01
2	Small polar	0.1275	**.14**	.00	.01	.03	.01	.02	.00	.03	.04	.03	.01	.03	.01	.02	.02	**.25**	**.28**	.07	.00	.01
3	Ambiguous	0.0030	.06	.06	.03	**.10**	.00	.04	.03	.02	.05	.06	**.06**	.02	.06	**.10**	.05	.06	.07	.05	.08	.00
4	Cysteine	0.0603	**.16**	**.36**	.01	.01	.01	.06	.01	.02	.01	.03	.01	.02	.02	.01	.02	**.09**	.07	.06	.00	.01
5	Aromatic	0.0532	.03	.01	.02	.02	**.08**	.03	.03	.01	.02	.03	.01	.02	.01	.02	.03	.03	.02	.02	**.46**	**.10**
6	Glyc or Pro	0.1441	**.18**	.00	.02	.02	.00	**.34**	.01	.01	.03	.01	.00	.01	**.24**	.01	.02	.05	.02	.01	.00	.00
7	Neg. charge	0.1415	.05	.00	**.21**	**.21**	.01	.06	.02	.01	.07	.02	.00	**.14**	.02	.05	.03	.06	.03	.01	.00	.01
8	Aliphatic	0.2137	.05	.01	.01	.01	**.11**	.01	.00	**.16**	.01	**.24**	**.06**	.01	.01	.01	.01	.01	.02	**.17**	.01	**.08**
9	Large polar	0.0857	.04	.00	.01	.02	.05	.02	**.43**	.01	.03	.03	.01	.04	.01	.04	.04	.04	.03	.02	.00	**.11**

### Scoring function

Each I-sites motif contains a two-part scoring function consisting of a position-specific profile and a pairwise covariant scoring function. Both were calculated from clusters of segments of length *L *from the database described above. The motif lengths 3 ≤ L ≤ 19 and structures were chosen as described in the earlier work [2]. Each segment in a cluster has *L *positions, each having profile (Equation 1), a class and three backbone angles. The sub-sections below describe how the scoring matrices were calculated from the clusters.

For the pairwise covariant part of the score, two functional forms were initially explored, the correlation coefficient (*r*, Equation 2) and the log-likelihood ratio (*LLR*). The correlation coefficient *r *was found to be a better metric than *LLR *in preliminary studies involving the training and testing of selected motifs, especially the amphipathic helix motif. Although purely empirical, the choice of covariance metric made statistical sense. *LLR*, defined as the logarithm of the frequency of occurrence of two classes at two positions, divided by their expected random occurrence, can take extreme negative values when the number of observations is small. The correlation coefficient *r*, on the other hand, is bounded between -1 and +1, so that summing *r *distributes the contribution to the score evenly over all position pairs. By taking extreme values, LLR scores can be dominated by a single position pair. This imbalance could also have been remediated by carefully assigning pseudocounts, but we chose to avoid the additional parameter.

### Calculation of correlation tensor

The correlation between two random variables is typically expressed as the sum of the product of the deviation of each random variable from its mean divided by the standard deviation of each variable:

(5) $$corr(x,y)=\frac{\sum_{i}\left(x_{i}-\bar{x})(y_{i}-\bar{y}\right)}{\sqrt{\sum_{i}{\left(x_{i}-\bar{x}\right)}^{2}\sum_{i}{\left(y_{i}-\bar{y}\right)}^{2}}}$$

The correlation between amino acid classes at two positions in a motif was calculated by summing over all observations of that motif in the database. Each observation was expressed as a string of amino acid classes. For example, one observation of an alpha helix cap motif might have the following sequence of classes, using integers ranging from 1 to 9: 2281287926952. Each number in this sequence represents the class closest (Equation 4) to the single-position profile (Table 2) at that position.

To calculate the correlation values we first define a delta function, *δ*, as a Boolean value for a given observation, *n*:

(6) $$\delta_{k}^{i}(n)=\left\{ \begin{array}{l} 1\text{ if class }k\text{ is at position }i\text{ for observation }n\\ 0otherwise \end{array}\right.$$

Now each of the *N *observations of the motif is expressed as a 2-dimensional matrix of Boolean values, *δ*. The correlation between class *k *at position *i *and class *l *at position *j *was calculated by applying Eq 5 over all instances, *n*, of the motif.

(7) $$\sigma (i,j,k,l)=\frac{\sum_{n=1}^{N}\left(\delta_{k}^{i}(n)-{\bar{\delta }}_{k}^{i})(\delta_{l}^{j}(n)-{\bar{\delta }}_{l}^{j}\right)}{\sqrt{\sum_{n=1}^{N}{\left(\delta_{k}^{i}(n)-{\bar{\delta }}_{k}^{i}\right)}^{2}\sum_{n=1}^{N}{\left(\delta_{l}^{j}(n)-{\bar{\delta }}_{l}^{j}\right)}^{2}}}$$

where $\bar{\delta }$ is the average Boolean property; i.e., ${\bar{\delta }}_{k}^{i}=\frac{1}{N}\sum_{n=1}^{N}\delta_{k}^{i}(n)$.

### Correlation score

The 4-dimensional tensor of correlations for motif *h*, *σ*_h_, was used as a lookup table to score an observed sequence, using the Boolean matrix $\bar{\delta }$ (Equation 6). For example, the correlation score (*c_score*) for position *n *is calculated using positions *n *through *n*+*L*-1 as follows.

(8) $$\begin{array}{l} c\_score(n,h)=\\ \sum_{i=1}^{L-1}\sum_{k=1}^{9}\delta_{k}^{i}(n+i-1)\sum_{j=i+1}^{L}\sum_{l=1}^{9}\delta_{l}^{j}(n+j-1)\sigma_{h}(i,j,k,l) \end{array}$$

where *L *is the length of the structural motif and 9 is the number of classes.

### Profile score

A profile segment of a query was scored against a same-sized motif profile using the similarity score *S *(Equation 4), summed over the motif positions including two additional positions before and after the structural motif, thus from *n*-2 to *n*+*L*+1 where *L *is the length of the structural motif. This is the scoring function used in the previous I-sites work and was retained for direct comparison in this work.

(9) $$\begin{array}{l} p\_score(n,h)=\\ \sum_{i=-2}^{L+1}\sum_{a=1}^{20}\log\left(\frac{P_{n+i,a}^{query}+\alpha F_{a}}{(1+\alpha )F_{a}}\right)\log\left(\frac{P_{i,a}^{h}+\alpha F_{a}}{(1+\alpha )F_{a}}\right) \end{array}$$

where *α *= 0.5, and *h *is the I-sites motif.

Typically, the similarity of two profiles is scores by calculating the cross entropy, Σ(*P*^*query*^)log(*P*^*h*^), which expresses the log of the joint probability of two distributions. Equation 9, *p_score*, however, is a correlation score over LLRs, which does not translate directly to a joint probability but has some of the same flavor. The difference lies in how low probabilities are scored. The Shannon entropy score ignores near-zero terms, but for *p_score *they contribute positively if both P^query ^and P^h ^are small, and negatively if one is small and the other large. If a motif profile has a significantly underrepresented amino acid in it, then it stands to reason that the presence of that amino acid signals negatively about the presence of the motif structure. As reported previously[2], we believe that the absence of certain amino acids at certain positions plays an important role in local folding.

### Linear combination of scores

For each of the I-sites motifs, *h*, the score *I_score(n,h) *was defined to be a linear combination of the profile score, *p_score *(Equation 9) and the correlation score *c_score *(Equation 8). A binary search in the range 0 ≤ *ω*_h _≤ 1 was used find the optimal relative weights, *ω*_h_.

(10) $$\begin{array}{l} \text{I\_score}(\text{n},\text{h})=\\ \omega_{\text{h}}\text{ p\_score}(\text{n},\text{h})+(1-\omega_{\text{h}})\text{c\_score}(\text{n},\text{h}) \end{array}$$

Each segment *n *has the additional property of being either a true or a false instance of the motif, based on the MDA metric. The value being optimized was the Receiver Operator Characteristic (ROC) curve [12]. The area under the ROC curve is a good overall metric of accuracy; a perfect classification method will have an area under the curve of one and a random binary classifier will have an area of approximately 0.5. Motif weights *ω*_h _were optimized one at a time using a binary search.

### Validation

The value of the I-sites library as a predictor of local structure was assessed by making predictions on a test set of 559 proteins not used in training, as described in the following sections. A set of 2249 proteins from the latest PDBSelect25 [15] was used to train the model. [For more information on how the datasets were constructed, see Additional File 1]

### Motif paradigm structures

As in previous I-sites work, each motif had a representative structure (*paradigm*) from the database, which was the structurally most central peptide in a set of clustered sequence segments. The paradigms were originally chosen as the peptide with the smallest sum of the root-mean-square-deviation (RMSD) to the other members of the cluster, and were not changed in the current study. Where predicting the backbone angles using I-sites, the motif with the highest confidence value is selected from the library, then the predicted backbone angles are the backbone angles of the paradigm of that motif.

### Structural similarity metric MDA

Backbone angles deviations have been shown to correlate strongly with local RMSD of backbone atom positions, the presence of conserved sidechain contacts, and conserved backbone hydrogen bonds. A maximum deviation over all backone angles (MDA) less than 120° corresponds to a maximum 1.4Å RMSD for an 8-residue fragment. Two segments, *r *and *s*, of length *L*, have the same structure (True) if the MDA passes a cutoff.

(11) $$MDA=\underset{i=2,L}{\max}\left[\left|\phi_{i}^{r}-\phi_{i}^{s}\right|,\left|\psi_{i-1}^{r}-\psi_{i-1}^{s}\right|,\left|\Omega_{i-1}^{r}-\Omega_{i-1}^{s}\right|\right]$$

Note that the *φ *angle of the first residue and the *ψ *and Ω of the last residue in the segment are ignored. The value of *cutoff *for each motif was chosen as its "natural boundary", or probability minimum, in previous work [2] and was not changed here. The average *cutoff *was 77°. Almost all segments that have MDA less than the natural boundary also conserve the motif 3D structure.

### Confidence assignment

The raw score, *I_score *(Equation 10) was converted to a probability, or *confidence *(*cf*) by fitting the score data to the accuracy data. The confidence reflects the probability that the sequence is correctly predicted to be the motif structure. [For details on how this was done, see Additional File 1]

### Overall accuracy estimation

The accuracy of local structure prediction was reported as the total percent correct by position, where correctness was assigned using the MDA metric (Equation 11). Note that overlapping correct predictions are not overcounted by this method since each position is counted exactly once. The expected value for random accuracy of a 8-residue segment prediction was found to be 5.7% by randomly choosing 8-residue pairs, using a generous *cutoff *= 120°. The same expected value was found after considering overlap, since random true predictions overlap rarely

### Training

Iterative supervised learning was carried out using expectation/maximization approach with a structure-based filtering step. The training set sequence segments assigned to a motif are referred to as a "cluster." The parameters of each motif, *P *(Equation 2), *σ *(Equation 7), and the confidence curve, are determined solely by the cluster membership. Thus only this membership was trained, by optimizing the fraction True (Equation 11) within the cluster. Motifs were trained independently.

#### Initialization

(1) Each length *k *segment in the training set was assigned a confidence score (*cf*) based on the motif profiles and confidence curve from the starting motif library, where *k *is the length of the motif.

(2) Each length *k *segment was assigned a True or False value using MDA (Equation 11).

(3) A new profile and tensor were summed using only the True segments (Equations 2,7).

#### Supervised learning

(4) Each length *k *segment in the training set was assigned scores *p_score *(Eq 9) and *c_score *(Equation 8):

(5) Each length *k *segment was assigned True or False (Equation 11).

(6) A linear combination of *p_score *and *c_score *(Equation 10) was found that maximized the ROC.

(7) A confidence curve was fit to the data.

(8) False segments and segments with confidence less than a cutoff (0.2) were pruned.

(9) The profile, *P*, and tensor, *σ*, were summed (Equations 2, 7).

(10) Steps 4 – 9 were repeated until there was no improvement in overall accuracy.

### Three training strategies

To test the degree to which covariance contributes to improved prediction and/or over-fitting of the data, three strategies were tried when carrying out training of the I-sites Libraries (ISL). In the first case, only the profiles were trained, and covariance was not used (ISL5.1). In the second (ISL5.2), both the correlation tensor and the profile were recomputed at each training cycle (step 9 above). In the third case only the correlation tensor *σ *was updated; the profile *P *was initialized and fixed (ISL5.3). All training was done using the same training set to facilitate direct comparison of the three different trained libraries. A fourth library (ISL5.4) was constructed by taking the highest accuracy of the equivalent motifs from libraries ISL5.1, ISL5.2, and ISL5.3.

## Authors' contributions

CB: designed research, performed calculations, analyzed data, wrote paper. BW: performed calculations, wrote paper. All authors read and approved the final manuscript.

## Supplementary Material

Additional file 1 **Supplementary Data and Methods**. The following are included as Supplementary Data. (1) The contents and method for construction of the training and testing data sets. (2) The method for fitting the confidence curve. (3)The method for defining the nine amino acid profile classes. (4) Another case study, the amphipathic helix motif. (5) A more detailed analysis of the differences between the three training stategies. (6) The history of the I-sites Library and a short description of the web server. A sample of webserver output is shown. (7) A figure illustrating the supervised learning approach. Click here for file
