# Supplementary material for: Pairwise covariance adds little to secondary structure prediction but improves the prediction of non-canonical local structure
Source: BMC Bioinformatics. 2008 Oct 10;9:429. doi: 10.1186/1471-2105-9-429 (PMC2579440; doi:10.1186/1471-2105-9-429)
Supplement: Additional file 1 — Supplementary Data and Methods. The following are included as Supplementary Data. (1) The contents and method for construction of the training and testing data sets. (2) The method for fitting the confidence curve. (3)The method for defining the nine amino acid profile classes. (4) Another case study, the amphipathic helix motif. (5) A more detailed analysis of the differences between the three training stategies. (6) The history of the I-sites Library and a short description of the web server. A sample of webserver output is shown. (7) A figure illustrating the supervised learning approach. [file 1471-2105-9-429-S1.doc]

**Pairwise covariance adds little to secondary structure prediction but improves the prediction of non-canonical local structure.**

Christopher Bystroff^1^,, Bobbie-Jo Webb-Robertson^2^

^1^Departments of Biology and Computer Science, Center for Biotechnology and Interdisciplinary Studies, Rensselaer Polytechnic Institute, Troy NY, USA

^2^Computational Biology and Bioinformatics, Pacific Northwest National Laboratory, Richland, WA, USA

# S1 SUPPLEMENTARY METHODS

## S1.1 Training and testing data

Protein structures from the PDBselect database (Hobohm and Sander, 1994) (version Apr 2007) were used to generate a non-redundant sequence-structure database. These 2808 families are a subset of the PDBSelect25 list of non-redundant sequence families, having no more than 25% sequence identity between any two families. Families in the PDBSelect25 list were excluded if the parent structure was

not well determined, if it was an integral membrane protein, or if it contained a large number of disulfide bonds. Disordered loops were omitted in both training and validation. Gaps and insertions relative to the parent structure in aligned homolog sequences were ignored. Both X-ray and NMR structures were used. The dataset was randomly divided into training set of 2249 (Table S1) structures and a test set of 559 (Table S2).

PSI-BLAST (Altschul and Koonin, 1998) was used to search the *'nr'* database for homologous sequences and to build multiple sequence alignments. There was no attempt to prevent any GenBank sequence from aligning with more than one PDBselect structure, however such ‘common hits’ were detected by flagging each *nr* sequence with its PDBselect parent(s). If two proteins had a common hit, these proteins were labeled as "distant homologs" and were kept together, either as part of the training set or as part of the test set, but not both. Thus the test set contained no homologs, nor distant homologs, of the training set proteins. A list of the 2249 proteins used in training the model, and the 559 proteins used in testing are available upon request. The training set and test set are much larger than the set used for training and testing the I-sites library as it was last reported (Bystroff, et al., 2000), and include NMR structures which were previously excluded. A control prediction experiment using the previous library was done to assess the improvements using the new method.

Figure S1 illustrates the supervised learning algorithm as decreibed in the main text.

## S1.2 Confidence assignment

To convert the raw *I_score* (Equation 10 in main doculment) to a prediction confidence, all segments in the training set were binned by *I_score*; then the percent True, was determined for each bin. To make the "confidence curve" monotonic and smooth, an arctangent function was fit to the means of the bins (*x,y*) = (<*I_score>*, <*%*True>) using four parameters,

. (S1)

Parameters of the confidence curve, *b_0_, b_1_, b_2_*, and *b_3_* were found such that the sum of the squared residuals Σ(*cf*(*i*) *- y*(*i*))^2^ was minimized. Fitting was done using restrained conjugate gradient method, where *b_2_* and *b_3_* were restrained to values near 0.5 and 1.0 respectively, and *b_1_* was restrained to positive values. The confidence curve parameters for each motif were saved for use in assigning confidences for prediction. Figure S2 shows a typical fit. Motifs that failed a fitness test (less than 8 bins with y > 0.05) were omitted from further consideration

# S2 SUPPLEMENTARY RESULTS and discussion

**S 2.1 Amino acid profile classes**

Amino acid class profiles were derived by K-means clustering of single position profiles. Each class models a typical single position evolutionary history. The evolutionary history of a single position in a protein is a signature for its three-dimensional environment, and has been used successfully in improving alignment of distant homologs (Huang and Bystroff, 2006). Table 1 (main article) summarizes the chemical nature of the each of the nine classes. Class 8, for example, accounts for conserved aromatic positions. Class 5 represents positions that conserve other hydrophobics. Table S1 lists the amino acid classes, their sidechain characteristics and database abundance. Note that class 3 is a “catch-all” class that may account for poorly aligned positions. Classes are not discrete sets; some amino acids are observed in more than one class. For example, tyrosine is found in classes 5, 8, and 9, depending on whether it substitutes for an aromatic, a non-polar or a histidine. Surprisingly, the two most similar classes are 1 and 7, positive charge and negative charge. This shows that there are a large number of charged positions that tolerate a change in sign.

**S 2.2 Case studies in local sequence covariance**

S2.2.1 An amphipathic helix motif

As a proof of the principle that pairwise covariance statistics are rooted in energetics, we first looked for covariant interactions that were predictive of the common amphipathic helix motif (Segrest, et al., 1990). If a scoring function is predictive and based on robust statistics, then the largest numbers should appear in the positions that are known to contribute to helix stability (Figure S3). Positions separated by 3 or 4 positions along the sequence have sidechains that form hydrophobic contacts if they fall on the hydrophobic side of the helix, and they sometimes form salt-bridges if they fall on the hydrophilic side. Salt bridges are inherently pairwise interactions and cannot be adequately accounted for using positionally independent scoring.

The well-known “heptad repeat” motif of the alpha helix has the pattern, PxNxPxN, where P is a polar sidechain, N is non-polar and x is either, while excluding Gly and Pro at all seven positions. For the purposes of this discussion we will number these positions from 1 to 7, as they appear in Figure S3. The strongest covariances were found in the highly variable 'x' positions 2, 4 and 6. These positions do not have a conserved property, but do show covariant propensities. Profile class 8 (aliphatic) at position 2 correlates negatively with class 8 at position 4 on the other side of the helix, but correlates positively with class 8 at position 6. Positions 2 and 6 are in contact in an alpha helix, so a positive correlation between hydrophobic sidechains makes energetic sense. Positions 2 and 4 do not make contact in a helix, but would be in contact in a beta strand. Thus the negative correlation between class 8 at positions 2 and 4 makes sense in terms of negative design.

We observed only a very small additional positive correlation score when the two contacting polar sidechains in the polar 'P' positions had opposite charges, and a negative correlation when contacting positions had the same charge (both class 1 or both class 7). The correct signs of these correlations helped to validate the statistical method somewhat, but the weakness of the signal suggests that salt bridges are not strongly conserved in amphipathic helices, and implicitly that they do not contribute substantially to helix stability. This is consistent with molecular dynamics studies that showed that salt bridges contribute little to helical content in alanine-based peptides (Ghosh, et al., 2003). In fact, the statistics of amino acid conservation overall supports this conclusions. In our cluster analysis of single positions described in section S2.1. We found that sidechain ionization state is only weakly conserved in multiple sequence alignments. We conclude that the overall energetic contribution of an exposed salt-bridge is small relative to a hydrophobic contacts, and that the strength of observed covariances mirrors this heirarchy.

In studying Figure S3, if we focus on the correlations between class 8 (hydrophobic) positions, then a recurrent theme is observed. In the amphipathic helix motif , class 8 at position 2 correlates with class 8 at positions 5 and 6, but not at positions 4 and 7. On the other hand, class 8 at position 4 correlates with a class 8 at position 7 but not at positions 5 or 6. In other words, sidechains that are in contact in the helix have correlated hydrophobic preferences. It should be noted that such correlations are ignored in a profile-only model. The new model better characterizes the heptad repeat pattern as being an ensemble of many variants. There are 20 amphipathic helix patterns in the I-sites library, ranging in length from 3 to 15 residues and having different phases relative to the one shown here. The overall accuracy of helix prediction using the new I-sites library trained with covariance, as compared to the library trained only on profiles, increased from 70.2% to 78.4% ROC.

## S2.3 Comparison of the results of the three different training methods

The starting motif library, ISL 3.1 was reported (Bystroff, et al., 2000) along with HMMSTR. A hidden Markov model for local structure based on I-sites. HMMSTR is ISL 4, but was not assessed here because it carries additional information about motif adjacencies. The new libraries were trained on proteins within PDBselect25 (Table S2), using three different strategies. In the first case, only the profiles were trained, and covariance was not used (ISL 5.1). In the second (ISL 5.2), both the correlation tensor and the profile were recomputed at each training cycle. In the third case only the correlation tensor σ was updated; the profile *P* was initialized and fixed (ISL 5.3). All training was done using the same training set to facilitate direct comparison of the three different trained libraries. A fourth library (ISL5.4) was constructed by taking the highest accuracy of the equivalent motifs from libraries ISL 5.1, ISL 5.2, and ISL 5.3.

The previously reported I-sites library (ISL 3.1) was used in a form essentially unchanged since the original reporting. The only change made to this library was the addition of a confidence curve for each motif. At this step, of 293 motifs in this library, 9 failed a fitness test and were omitted.

Training eliminated some previously reported motifs. A motif was eliminated if it had no highest confidence predictions. This could happen if one of the re-trained motifs now recognized all or most of the members of another motif. In previous work, rare motifs were discovered by iteratively removing true positive predictions above a 70% confidence threshold from the training set, then re-clustering to find new patterns and training those patterns on the remaining part of the database. This procedure found significant sub-classes of motifs that would otherwise have been assigned to the more abundant class (Bystroff, et al., 2000). This procedure was omitted in the present study, since the goal was to assess the addition of covariance to the motif library. No new motifs were added to the library in this study.

S2.3.1 I-sites Library 5.1: Profile refined only.

As a control experiment in this attempt to demonstrate sequence covariance in local structure, the previous library was updated using only the profile information. The results show that updating the library greatly improved the overall prediction performance. Over the years since I-sites was originally trained, the size of the PDBselect25 database has grown by about 3-fold. Updating the profiles by iterative retraining led to improved accuracy across all local structure types.

S2.3.2 I-sites Library 5.2: Tensor and profile refined together.

The second training strategy was to update both the correlation tensor and the amino acid profile at each iteration The library 5.2 profiles after training varied in their appearance. Local structures such as alpha helix and beta strand with repeating Ramachandran angles evolved into relatively "flat" profiles while the correlation tensor increased in variance, adopting the greater share of the combined profile/correlation score. In retrospect, this is the expected result when training a profile on a structure with repeating backbone angles such as alpha helix and beta strand. Such structures match the motif structure in any shifted register. This promiscuity in alignment leads to the smearing of the amino acid probabilities over consecutive positions. On the other hand, some turn motifs, lacking a repeating pattern, aligned faithfully in one register and retained their sharply defined (low entropy) profiles. The observation that the profiles in library 5.2 were often lower in information content (higher entropy) than the profiles in the libraries 5.1 and 5.3 shows that pairwise correlations can replace the information in a profile and are often sufficient to predict the structure of a local motif. Motifs containing conserved glycines or prolines held more of the scoring function weight (Eq. 10) in the profile score than motifs that did not contain a glycine or proline. The energetic contribution of these turn-promoting residues is mostly entropic and is not captured by pairwise contact information. Furthermore, since they do not vary, they do not co-vary.

S2.3.3 I-sites Library 5.3: Tensor refined, profile fixed.

ISL 5.3, in which the tensor was iteratively updated but the profile was not, was found to have a lower prediction performance than both Libraries 5.1 and 5.2. By doing this second control experiment, we can see that the improvements in prediction accuracy for helix and strand motifs in version 5.2 are accounted for by the refinement of the profiles, as in library 5.1, not by the addition of the covariance tensor. However, we see improvements in accuracy in the prediction of most loop motifs, including helix caps. Again, a possible rationalle for this result is that loop motifs are anchored by certained required glycines and prolines, while the remaining contacts obey additive pairwise energetics. Beta strands and alpha helices are not anchored in this sense, but contain a repeating sequence pattern. A profile, as an all-or-none model, is a better predictor of a multiposition pattern.

The training process involved optimizing a relative weight of the correlation score and the profile. A relative weight was chosen that maximized the ROC for the predictions of one motif. For ISL 5.2, the average weight ratio (the correlation tensor weight, 1-*w_h_*, divided by profile weight, *w_h_*), averaged over all motifs, was 2.4, whereas this ratio for library 5.3 was 1.7. The optimal combination of correlation and profile scores had placed more weight on correlation when the two were trained together, compared to when they were trained apart. Along with the observations of flattened profiles in library 5.2, the changed weight balance supports the conclusion that the four-dimensional correlation tensor effectively subsumes all of the information content of the two-dimensional profile during the course of iterative refinement. But while we gained senstivitity to pairwaise covariance, we lost the multibody component of the motif model.

We conclude from this experiment that, in canonical secondary structure, the profile and the correlation tensor are both being fit to a multivariant sequence pattern, which is better captured by a profile of 20 amino acids than a tensor of 9 classes. For non-canonical local structure motifs, the two scoring functions have less overlap, and the effect is that the combined scoring function is better than a profile. It is possible that a different scoring metric for covariance would better capture the observed covariant patterns.

# S3 history and availability

Each new version of the I-sites library (ISL) has received a new version number since 1997. ISL 1 was used in CASP2 predictions (Bystroff and Baker, 1997). ISL 2 introduced 2-residue motif extensions and iterative pruning and re-clustering of the protein structure database as described in (Bystroff & Baker, 1998). ISL 3, into which "rare motifs" were added by iterative removal and re-clustering, was described in (Bystroff, et al., 2000) ISL 4 was reformulated into a hidden Markov model called HMMSTR (Bystroff, et al., 2000). ISLs 5.1, 5.2 and 5.3 are described here. ISL 4, or HMMSTR, was not used in this comparison since it would have introduced the Markov property. Unfortunately we do not know how to create a covariant HMM.

The latest I-sites libraries are available online (http://www.bioinfo.rpi.edu/bystrc/Isites2/). Older libraries are available upon request. I-sites Library 5.2 has replaced Library 3.1 as a tool in the I-sites/HMMSTR/Rosetta server (www.bioinfo.rpi.edu/bystrc/hmmstr/server.php). Users receive a hyperlinked image of fragment predictions as a function of position and prediction confidence (Figure S4).

# S4 SUPPLEMENTARY REFERENCES

Altschul, S.F. and Koonin, E.V. (1998) Iterated profile searches with PSI-BLAST--a tool for discovery in protein databases, *Trends in biochemical sciences*, **23**, 444-447.

Bystroff, C. and Baker, D. (1997) Blind predictions of local protein structure in CASP2 targets using the I-sites library, *Proteins*, **Suppl 1**, 167-171.

Bystroff, C., Thorsson, V. and Baker, D. (2000) HMMSTR: a hidden Markov model for local sequence-structure correlations in proteins, *Journal of molecular biology*, **301**, 173-190.

Ghosh, T., Garde, S. and Garcia, A.E. (2003) Role of backbone hydration and salt-bridge formation in stability of alpha-helix in solution, *Biophysical journal*, **85**, 3187-3193.

Hobohm, U. and Sander, C. (1994) Enlarged representative set of protein structures, *Protein Sci*, **3**, 522-524.

Huang, Y.M. and Bystroff, C. (2006) Improved pairwise alignments of proteins in the Twilight Zone using local structure predictions, *Bioinformatics (Oxford, England)*, **22**, 413-422.

Segrest, J.P., De Loof, H., Dohlman, J.G., Brouillette, C.G. and Anantharamaiah, G.M. (1990) Amphipathic helix motif: classes and properties, *Proteins*, **8**, 103-117.

**Figure S1.** Supervised learning. Clockwise from the upperleft: A cluster of similar structures is used to generate, a profile and correlation tensor which are used to find, nearest neighbor sequences in the database which are, superimposed in 3D and then filtered against a paradigm structure to give, a cluster of similar structures, and so on.


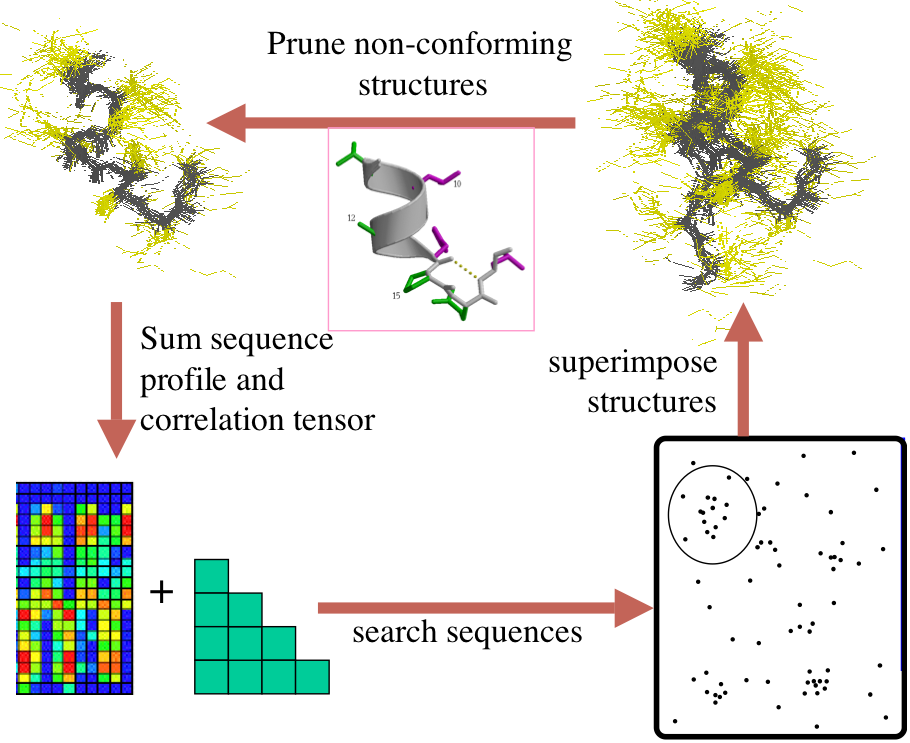


**Figure S2.** **-** Confidence curve. Each dot represents 200 scores in a sorted list of all scores for all segments within the PDBselect25 training set, plotted by motif score and percent true. The curve represents the least squares fit, relating the motif score to the confidence.


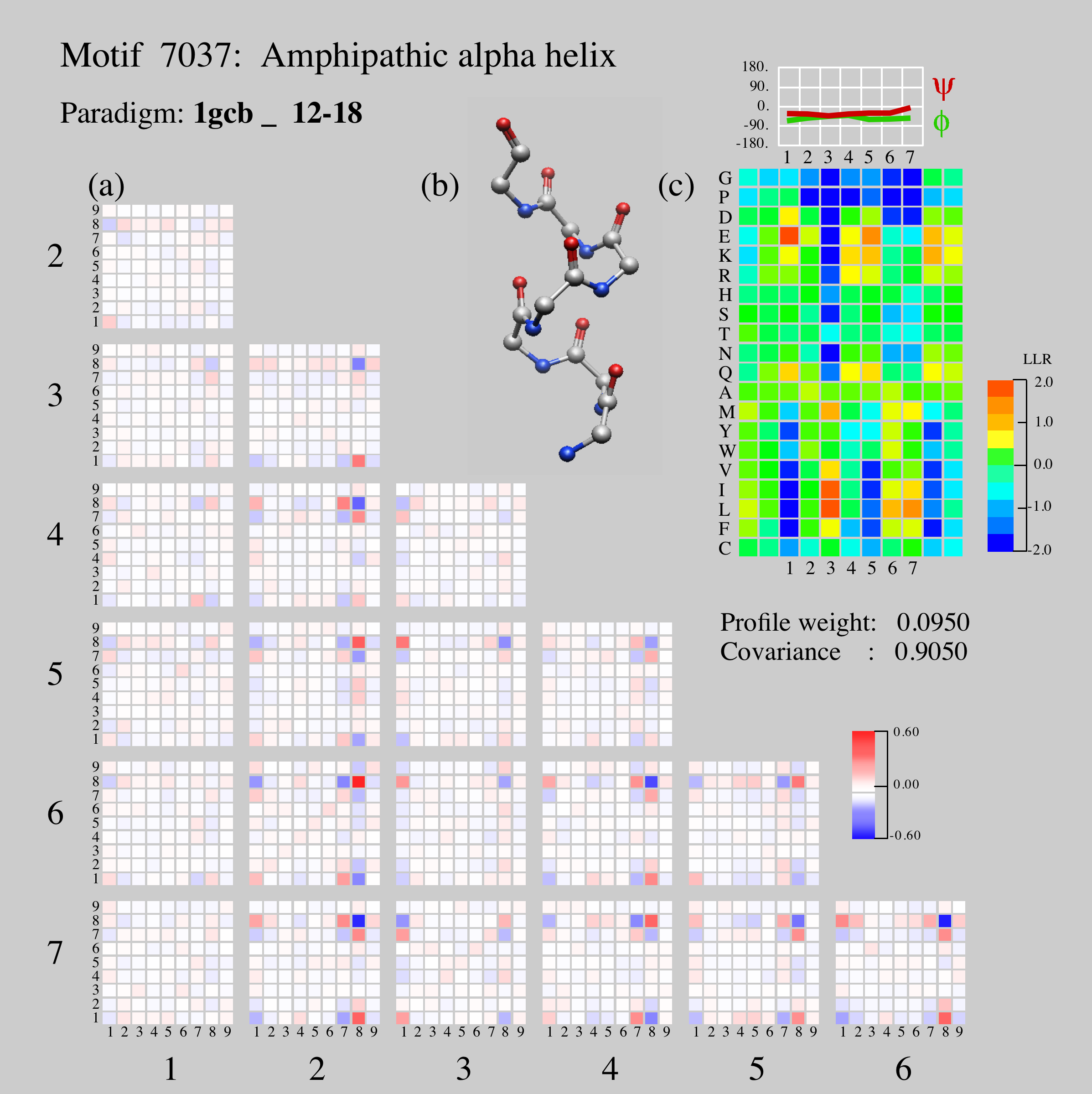


**Figure S3**. Amphipathic alpha helix from ISL5.2. (a) Correlation tensor. Large numbers indicate positions in the motif, small numbers indicate amino acid class. Correlation is expressed as a color from blue to red according to the scale in the lower right. (b) ball-and-stick rendering of the motif structure. (c) Profile expressed as log-likelihood ratios according to the color scale at the right. Amino acids are arranged roughly from non-polar on the bottom to polar on the top. Plotted above are backbone angles for each position.


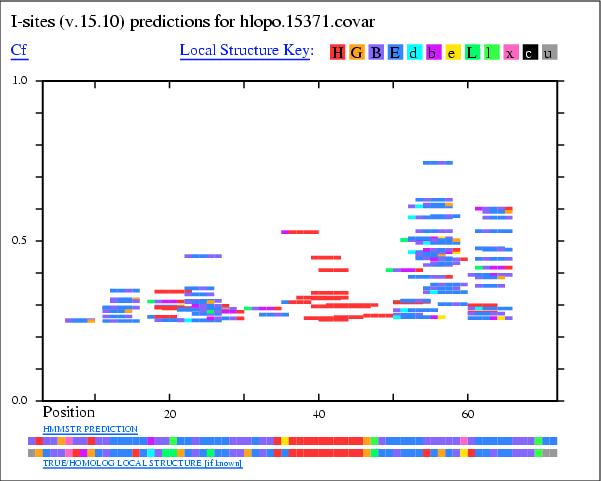


I-sites v.5.2 predictions for

**Figure S4**. User output for I-sites from HMMSTR server. Bars mark positions, length and confidence of motif predictions. Colors in bars represent backbone angle regions for each residue.

**Table S1.** Training set proteins from PDBselect25, version Jan 2007. PDB code plus chain identifier, if present. There are a toal of 2249 chains.

1a02F 1a0aA 1a1x 1a2n 1a56 1a5oA 1a63 1a6bB 1a6m 1a8y 1a92A 1aa3 1ab3 1aduA 1adwA 1afoA 1ag4 1aho 1aihA 1aiw 1ajj 1ako 1alu 1aly 1aohA 1aojA 1aol 1aps 1aq4A 1aq5A 1aqzB 1at0 1atx 1auxA 1avoA 1axh 1ayj 1ayoA 1ayyB 1az6 1b04B 1b0nA 1b1a 1b26A 1b33N 1b35D 1b4g 1b4uB 1b52A 1b55B 1b5eA 1b6fA 1b8mB 1b8wA 1b9qA 1b9uA 1b9xA 1ba4 1bak 1bb1A 1bb8 1bby 1bcrA 1bcrB 1bdo 1bea 1bev3 1bg8A 1bgf 1bgk 1bh4 1bh7 1bh8B 1bh9A 1bj8 1bk6A 1bkrA 1bl1 1bm4A 1bnlA 1bnxA 1bp3A 1bplA 1bplB 1bqcA 1bqhG 1br9 1brz 1bshA 1bt6A 1bu2A 1bu7A 1bu9A 1bwsA 1bx7 1bxdA 1bxnI 1byi 1bywA 1c01A 1c05A 1c07A 1c1kA 1c28A 1c4rG 1c5eA 1c5lL 1c6wA 1c75A 1c7kA 1c8oA 1c9iA 1c9oA 1c9uB 1cawB 1cc8A 1ccvA 1ce4A 1ceuA 1cf4B 1cf7A 1cf7B 1cfh 1chvS 1ci0B 1ci4A 1ci5A 1cid 1cirA 1cixA 1cjgA 1ckv 1cl3A 1clvI 1cmbA 1cmr 1co4A 1cokA 1copD 1couA 1cpzA 1cqjB 1cqmA 1cseI 1ctj 1cv8 1cvrA 1cw5A 1cwxA 1cxqA 1cxwA 1cy5A 1cyl 1cz4A 1czpA 1d0dA 1d0qA 1d0rA 1d1nA 1d2hA 1d2pA 1d2zB 1d3bA 1d3bH 1d4oA 1d4uA 1d5tA 1d6gA 1d6mA 1d7pM 1d8bA 1d9aA 1d9uA 1danT 1dcfA 1dchA 1dcs 1ddbA 1ddmA 1ddwA 1de5A 1devB 1df4A 1dfmA 1dfnA 1dguA 1dgzA 1dipA 1dj7A 1dj7B 1dk2A 1dk4A 1dkcA 1dmlB 1dnyA 1domA 1dosA 1dp3A 1dp7P 1dqcA 1dqeA 1dqtA 1dqzA 1ds1A 1dsvA 1dszB 1dtdB 1dujA 1dxmA 1dxwA 1dxzA 1dzkA 1e0gA 1e0jA 1e0lA 1e2aA 1e2b 1e44A 1e4tA 1e6iA 1e7lA 1e8qA 1e9gA 1e9kA 1eaiC 1eajA 1eaqB 1eayC 1eb0A 1eb6A 1ecbA 1ecd 1eciA 1ecrA 1ecsA 1ecxA 1edsA 1edvA 1edxA 1ef1C 1ef4A 1ef5A 1eggB 1egwA 1egxA 1ehs 1ehyA 1ei0A 1eiwA 1ejgA 1ekzA 1el6A 1elkA 1em2A 1emwA 1enwA 1eo1A 1ep2B 1eq1A 1eqa 1equA 1erd 1es5A 1es7D 1es9A 1et1A 1eteD 1euvA 1euwA 1ev0A 1ew4A 1ewiA 1ewsA 1excA 1exh 1eyhA 1eyrB 1ezeA 1ezgA 1f02T 1f0cB 1f0zA 1f1eA 1f32A 1f3oA 1f3uD 1f45A 1f4rA 1f51B 1f53A 1f5eP 1f5qD 1f60B 1f7lA 1f81A 1f86A 1f8eA 1f94A 1f9vA 1fadA 1fafA 1faq 1fbr 1fbtA 1fbvA 1fcvA 1fe8C 1feuD 1fexA 1fhoA 1fiqB 1fit 1fj9A 1fjcA 1fjgN 1fjpA 1fjrA 1fkmA 1fl7D 1fleI 1fliA 1flmA 1fltY 1foeB 1foy 1fqjC 1fqkC 1fqqA 1fr2A 1fr6A 1fre 1frwA 1fshA 1ft4A 1fujA 1fuoA 1fuwA 1fvkA 1fvpA 1fwoA 1fxkC 1fyeA 1fywA 1fzeC 1fzeD 1fzpB 1fztA 1g11A 1g1pA 1g25A 1g2bA 1g2cH 1g3p 1g43A 1g4gA 1g4yB 1g55A 1g5mA 1g5qA 1g61A 1g66A 1g6uA 1g6xA 1g6zA 1g7eA 1g84A 1g8aA 1g8qB 1ga3A 1ga6A 1gakA 1gc6A 1gccA 1gcf 1gci 1gcqC 1gd0A 1gd4A 1gdf 1gefD 1gh1A 1gh5A 1gh9A 1ghc 1ghhA 1ghk 1gjxA 1gk7A 1gk9B 1gkmA 1gl2A 1gl2C 1gl2D 1gl4B 1gllO 1gmxA 1gnf 1gnhA 1go1A 1goeA 1gp0A 1gpiA 1gps 1gqwB 1gtpA 1gtqA 1gu2A 1gu6A 1guoA 1guxB 1gv9A 1gvdA 1gvmA 1gvnD 1gvp 1gw4 1gw5S 1gweA 1gwmA 1gxbD 1gxmA 1gxrA 1gxuA 1gxyA 1gya 1gyxA 1gyzA 1h03P 1h0tB 1h1cB 1h1jS 1h29D 1h2cA 1h2nA 1h2wA 1h32A 1h3qA 1h3zA 1h4aX 1h4lD 1h4uA 1h4xA 1h6oA 1h75A 1h7cA 1h7dA 1h7yA 1h8cA 1h97A 1h9eA 1ha8A 1hce 1hcfX 1hcrA 1hd6A 1hdlA 1hdoA 1hf2D 1hf9A 1hfaA 1hfi 1hg4C 1hi8A 1hj0A 1hkqA 1hkyA 1hl6D 1hl9A 1hlgA 1hlm 1hlqA 1hm7B 1hmjA 1hn3A 1hn6A 1hns 1hofA 1hq6A 1hq6B 1hqbA 1hqkA 1hqoA 1hruB 1hs5A 1htrP 1htwA 1huxA 1hwhB 1hx0A 1hx2A 1hxrA 1hykA 1hyoA 1hz6B 1hztA 1i0dA 1i0vA 1i17A 1i18A 1i1aC 1i1wA 1i26A 1i27A 1i2tA 1i2uA 1i2zA 1i3sC 1i4vA 1i4zA 1i6zA 1i71A 1i78B 1i8aA 1i8oA 1i9bA 1iad 1iajB 1iarB 1ib1E 1ib9A 1ibyA 1id1B 1idpA 1ie9A 1ifqA 1ig4A 1igl 1ignA 1iguB 1iijA 1iioA 1iisC 1ij9A 1ijyA 1ilvA 1im0A 1im3D 1imt 1in1A 1ioiA 1ipgA 1iqzA 1irdA 1irl 1irsA 1iruC 1iryA 1irzA 1is3A 1is7K 1ismA 1it2A 1itf 1itqA 1iu9A 1iuaA 1iufA 1iujA 1iuyA 1iv3A 1ivzA 1iwcA 1ix2A 1ix5A 1ix9A 1ixh 1iycA 1iz1B 1j03A 1j0pA 1j0sA 1j0tA 1j1vA 1j2rA 1j31A 1j3gA 1j5lA 1j6rA 1j75A 1j7rA 1j7vL 1j7vR 1j98A 1j9aA 1j9iA 1jatB 1jb0J 1jb0M 1jb9A 1jbeA 1jbiA 1jc5A 1jcdA 1jcqB 1jd2L 1jdhB 1jdmA 1je5B 1jeiA 1jekA 1jeoA 1jf8A 1jfbA 1jh5A 1jh6A 1jhgA 1jhjA 1ji3A 1ji7A 1jidA 1jjdA 1jk9B 1jkuA 1jkw 1jl0B 1jl1A 1jl7A 1jli 1jm1A 1jmtA 1jmvA 1jn0A 1jn7A 1jnzB 1jo6A 1jogD 1jopA 1jr5A 1jr8B 1jruA 1jsuC 1jumA 1junA 1jvr 1jw3A 1jwqA 1jx7A 1jxcA 1jy2P 1jy2R 1jyaA 1jybA 1jyoA 1jytA 1k0hA 1k0mA 1k1fE 1k1qB 1k2fA 1k2nA 1k3bA 1k3iA 1k3kA 1k3xA 1k40A 1k4uP 1k55A 1k5cA 1k5kA 1k5nA 1k5nB 1k5oA 1k7cA 1k7jA 1k85A 1k92A 1k94A 1k9cA 1ka1A 1ka8A 1kafA 1kbhA 1kcgC 1kcqA 1kejA 1kf6B 1kf6C 1kf6D 1kg1A 1kgdA 1khoA 1khyA 1kicA 1kilE 1kjlA 1kjs 1kldA 1klrA 1klxA 1kmvA 1kn0A 1kn6A 1knmA 1knzB 1kotA 1kp0A 1kpsB 1kptA 1kq5A 1kq6A 1kqfB 1kqfC 1kqhA 1kqpA 1kr2F 1kr4A 1ks8A 1ksjB 1ksqA 1ksr 1ksvA 1kthA 1ktuA 1kufA 1kum 1kutA 1kv7A 1kvdA 1kvdB 1kwaA 1kwfA 1kwiA 1kxrA 1kyfA 1kyoH 1l0oA 1l1cA 1l1oA 1l1sA 1l3gA 1l3kA 1l3lA 1l3pA 1l3yA 1l4aA 1l4aC 1l5iA 1l6l5 1l6xB 1l8cB 1l8oA 1l8rA 1l9lA 1l9vA 1l9yA 1la3A 1lc7A 1lddA 1ldjB 1lhvA 1liaA 1lj9B 1ljoA 1lknA 1ll8A 1llfA 1llqA 1lmiA 1lmmA 1ln2A 1lnuA 1lo7A 1lpbA 1lpvA 1lq1B 1lq9A 1lqtA 1lr0A 1lr1A 1lrtB 1ls1A 1lslA 1ltzA 1lugA 1lupA 1luzA 1lv3A 1lv9A 1lwbA 1lxjA 1ly7A 1lyp 1lzwA 1lzwB 1m0kA 1m15A 1m1fA 1m1nA 1m1nB 1m1qA 1m2dA 1m2fA 1m2iA 1m36A 1m40A 1m4lA 1m4pA 1m56D 1m57C 1m5zA 1m7eA 1m7gB 1m9xC 1m9zA 1maeL 1mavA 1mbyA 1mc2A 1mdyB 1me4A 1me5A 1mf7A 1mfmA 1mg4A 1mg7A 1mgsA 1mhdA 1mhzB 1mhzG 1mi8A 1mj5A 1mk0A 1mk2A 1mkcA 1mkdA 1mkiA 1mkkA 1mknA 1mm3A 1mm4A 1mml 1mntA 1moq 1mp1A 1mr2A 1mr8A 1mr9C 1mruB 1msoB 1muwA 1muzA 1mv4A 1mvxA 1mwbA 1mwjA 1mwpA 1mwqA 1mwwA 1mzbA 1mzgB 1mzwB 1n0wB 1n0zA 1n13A 1n13B 1n1dA 1n1hA 1n32J 1n3gA 1n4dA 1n4nA 1n4wA 1n55A 1n5gA 1n62A 1n62C 1n69A 1n6uA 1n6zA 1n7gC 1n7iB 1n7sB 1n7sD 1n8kA 1n8vA 1n9pA 1nayA 1nblA 1nc5A 1ncnA 1ncs 1nd9A 1ne3A 1ne8A 1neiA 1nenD 1neq 1new 1nf9A 1ng7A 1ngmB 1ngnA 1nh0A 1nh2B 1nhyA 1nivA 1nixA 1njjA 1njqA 1nkiA 1nkpD 1nkzA 1nkzB 1nlqA 1nls 1nlxA 1nlzE 1nmpA 1nn5A 1nnuC 1nofA 1npyB 1nqjA 1nrjA 1nrjB 1nrxA 1ns1A 1nskL 1nvjA 1nvoA 1nw1B 1nw8A 1nwbA 1nwnA 1nwwA 1nwzA 1nxiA 1nxmA 1ny71 1nycA 1nynA 1nz0A 1nz9A 1o08A 1o0pA 1o2dA 1o2fB 1o4tA 1o4uB 1o50A 1o5pA 1o67A 1o6aA 1o6bA 1o7iB 1o7lD 1o7qA 1o8rA 1o8tA 1o9rA 1o9vA 1oaa 1oaiA 1oapA 1oayO 1obdA 1oboA 1ocyA 1oczD 1oczE 1oczF 1odgA 1odhA 1odmA 1oeyC 1ofnA 1ofqB 1og6C 1og7A 1oh1A 1oh4A 1ohzB 1oi0D 1ojgA 1ojhD 1ojrA 1ok0A 1okcA 1ol1B 1oo5A 1oocA 1oopA 1op4A 1opd 1oq9A 1oqeK 1oqvA 1or6A 1orhA 1osyA 1otgA 1otrA 1ovqA 1ovyA 1ovzA 1owtA 1oxdA 1oyjD 1p0zA 1p16B 1p1mA 1p1xB 1p32B 1p3bC 1p3qQ 1p4cA 1p4lD 1p4qA 1p5cA 1p5zB 1p6oA 1p7nA 1p88A 1p8bA 1p94A 1p9gA 1p9kA 1pb5A 1pb7A 1pba 1pbjA 1pbuA 1pbyC 1pc6B 1pcfA 1pcp 1pd3A 1pdc 1peh 1pex 1peyB 1pfbA 1pfsA 1pfvA 1pgs 1pgyA 1ph7A 1pho 1pih 1pil 1pinA 1pjxA 1pkhA 1pmhX 1pms 1pp0A 1pp7U 1pq1B 1pqhA 1pqsA 1pqwA 1prtD 1prtF 1psrA 1psyA 1ptq 1pulA 1puoB 1puzA 1pv1A 1pv2B 1pvzA 1pwbA 1pxqA 1pybA 1pyoB 1pytA 1pyvA 1pz4A 1pzdA 1pzqA 1pzwA 1q01A 1q0dA 1q1vA 1q2hB 1q2iA 1q3iA 1q3zA 1q4kC 1q53A 1q5wA 1q5yD 1q68A 1q6wG 1q7lC 1q86J 1q8bA 1q8dA 1q8rB 1q92A 1q97A 1qa4A 1qa7B 1qc7B 1qc9A 1qczA 1qddA 1qdp 1qeyA 1qfcA 1qg7A 1qgkB 1qgmA 1qgvA 1qh9A 1qhhB 1qhhC 1qhkA 1qj4A 1qkqA 1ql0A 1qldA 1qleA 1qleB 1qloA 1qlwA 1qmcA 1qmpA 1qmtA 1qnlA 1qnrA 1qo0E 1qo4A 1qolC 1qopB 1qouB 1qp2A 1qp6A 1qpmA 1qqeA 1qqfA 1qqhA 1qqiA 1qr0A 1qsoA 1qtnB 1qtwA 1qu9A 1qvpA 1qw2A 1qwoA 1qxnA 1qynD 1qz4A 1r02A 1r0bG 1r0rI 1r0uA 1r2aA 1r2cM 1r2mA 1r3bA 1r4aE 1r4gA 1r4pA 1r5lA 1r5sA 1r5tB 1r5yA 1r61A 1r6aA 1r6jA 1r6yA 1r77A 1r79A 1r7gA 1r7jA 1r8hA 1r8nA 1r9fA 1ra0A 1rbmA 1rc8A 1rdqE 1regY 1res 1rfgE 1rfhA 1rfvA 1rgoA 1rh6A 1rhfA 1rhqA 1ri0A 1rip 1rjlC 1rjuV 1rkeB 1rkiB 1rklA 1rkuA 1rmd 1rmkA 1ro2A 1ro5A 1ro8A 1roo 1rq6A 1rqrA 1rqwA 1rsoA 1rsoB 1rtqA 1rttA 1rtyA 1rutX 1rw5A 1ry9A 1ryjA 1rykA 1ryoA 1rypL 1rz2A 1rz4A 1rzsA 1s0pA 1s0wC 1s1cY 1s2eA 1s2xA 1s3aA 1s3tB 1s4yC 1s4zC 1s5uG 1s6dA 1s6xA 1s89A 1s99B 1s9uA 1sauA 1sawA 1saxA 1sb6A 1sboA 1sbxA 1sc3B 1scjB 1scmA 1scy 1sezA 1sf0A 1sf8H 1sfdA 1sfjA 1sfp 1sfuA 1sfxB 1sg3B 1sg5A 1sgoA 1shi 1shuX 1shwB 1si6X 1sis 1sj1A 1sj5A 1sjwA 1sk3A 1skvC 1sm7A 1smpI 1sn8B 1snlA 1sp3A 1sqrA 1sr2A 1sr4A 1sr4C 1srkA 1ss3A 1sseA 1sslA 1ssxA 1stmA 1sto 1su8A 1suyA 1sv1C 1svfB 1sxiA 1syyA 1szhB 1szvA 1t0cA 1t0fC 1t0pB 1t0rC 1t13D 1t15A 1t17A 1t1hA 1t1jB 1t1uA 1t1vA 1t23A 1t2dA 1t2kB 1t2tA 1t33A 1t34B 1t3oA 1t4cB 1t4lB 1t50A 1t5jA 1t5qA 1t5yA 1t61B 1t62A 1t6fA 1t6sA 1t6yB 1t75A 1t7rA 1t84A 1t8iA 1t92A 1t9iB 1tafA 1tbaB 1tbuC 1tc0B 1tdqA 1teyA 1tf1B 1tfb 1tfe 1tfi 1tfmB 1tfoB 1th5A 1th7A 1thjA 1ti8B 1tiiD 1tikA 1tiyA 1tjyA 1tknA 1tl2A 1tle 1tlhB 1tljB 1tltB 1tm0A 1tm9A 1tme1 1tnrA 1to0B 1totA 1tp8A 1tpn 1tq1A 1tq6A 1tqgA 1tqwA 1tt8A 1ttuA 1ttwA 1tu1A 1tu9A 1tueK 1tukA 1tuzA 1tv0A 1tv7A 1tvs 1twcI 1twcK 1twwB 1txm 1ty2A 1tykA 1tyqE 1tz0A 1tzpB 1tzvA 1u02A 1u07A 1u0sA 1u2tA 1u2xA 1u3nA 1u3oA 1u5mA 1u61A 1u69D 1u6lA 1u81A 1u84A 1u8sB 1ua8A 1ub4B 1ub9A 1ucdA 1ucpA 1ucsA 1ucvA 1uexB 1ufbA 1ufmA 1ufqD 1ufyA 1ug1A 1ug2A 1ug6A 1uglA 1ugqA 1uilA 1ujdA 1ujoA 1ujtA 1ujvA 1ujwB 1uk1A 1uk5A 1uklC 1umiA 1uowA 1uoyA 1uphA 1uq5A 1uqvA 1ur6B 1urhA 1us0A 1us1A 1uscA 1useA 1usmA 1ussA 1usyC 1ut3A 1ut4A 1uutA 1uv4A 1uvfA 1uw0A 1uw1A 1uw2A 1uw4A 1uw5A 1uwkB 1uwwB 1uxaA 1uxoA 1uz0A 1uz3A 1uzcA 1uzxA 1v06A 1v0wA 1v14D 1v1cA 1v1qA 1v2bB 1v2xA 1v2yA 1v30A 1v31A 1v32A 1v38A 1v3aA 1v3fA 1v4pA 1v54G 1v54H 1v54I 1v54J 1v54K 1v54L 1v5iB 1v5kA 1v5lA 1v5rA 1v63A 1v65A 1v6pA 1v70A 1v7uB 1v83A 1v85A 1v8hA 1v92A 1v95A 1v9vA 1va1A 1va2A 1vazA 1vbwA 1vcbC 1vcc 1vchE 1vcsA 1vd4A 1vd6A 1vdxA 1vdzA 1vegA 1vehA 1veuA 1vf5S 1vfiA 1vhh 1vhiB 1vhuA 1vhwE 1vi8B 1vib 1vizB 1vjfA 1vjhA 1vjnA 1vjqA 1vjuA 1vjxA 1vjzA 1vk1A 1vkbA 1vkpB 1vkwA 1vldN 1vmgA 1vnd 1vpaB 1vpu 1vpxG 1vpyA 1vqo2 1vqo3 1vqsA 1vr8A 1vrlA 1vrsB 1vyiA 1vyqA 1vyrA 1vyxA 1vzsA 1vzv 1w09A 1w0cC 1w0eA 1w0hA 1w1hC 1w1nA 1w23B 1w2eA 1w2qA 1w2wB 1w2yB 1w3dA 1w3gA 1w4sA 1w66A 1w6sB 1w8aA 1w8xM 1w9rA 1wa8A 1wa8B 1wazA 1wb4A 1wbc 1wbeA 1wc4B 1wclA 1wcwA 1wd2A 1wdvA 1we1A 1we9A 1welA 1weoA 1wf6A 1wffA 1wfjA 1wfkA 1wfqA 1wfrA 1wftA 1wgdA 1wgfA 1wgkA 1wgmA 1wgnA 1wgvA 1wgwA 1wgxA 1wgyA 1wh5A 1wh6A 1wh9A 1whgA 1whi 1whqA 1whrA 1whuA 1whxA 1whzA 1wi9A 1widA 1wigA 1wiiA 1wimA 1wirA 1wizA 1wj0A 1wj2A 1wj7A 1wjiA 1wjjA 1wjtA 1wjvA 1wjzA 1wkiA 1wkt 1wloA 1wluA 1wm3A 1wmhA 1wmhB 1wmiA 1wmvA 1wmyA 1wn2A 1wotA 1wpiA 1wpkA 1wprA 1wpuA 1wq6A 1wqlB 1wr8A 1wrgA 1wrjA 1wrvC 1wspB 1wsuC 1wsvA 1wsxA 1wt8A 1wtdA 1wthD 1wtuA 1wu0A 1wuiS 1wuzA 1wvfA 1wvqA 1wvzA 1wwqA 1wwtA 1wwuA 1wxcA 1wxlA 1wxmA 1wxnA 1wxpA 1wy3A 1wy6A 1wyzC 1wz3A 1x0oA 1x0tA 1x22A 1x32A 1x3aA 1x3wA 1x49A 1x4yA 1x58A 1x5wA 1x62A 1x6fA 1x6jA 1x6oA 1x82A 1x8dA 1x91A 1x99B 1x9dA 1x9rB 1xa7A 1xapA 1xaxA 1xbd 1xbiA 1xbwA 1xc0A 1xc5A 1xcbG 1xdnA 1xdqA 1xdxA 1xe8B 1xeeA 1xeoA 1xeqA 1xer 1xexA 1xfkA 1xfoA 1xg0A 1xg0C 1xg4D 1xhbA 1xhhA 1xizA 1xj5B 1xjlA 1xjsA 1xjuB 1xkeA 1xkpC 1xkrA 1xksA 1xm0A 1xmeC 1xmkA 1xmtA 1xn5A 1xn7A 1xn9A 1xo1A 1xoyA 1xpjA 1xpnA 1xppA 1xpvA 1xpxA 1xq4C 1xqaB 1xqoA 1xqxA 1xr0B 1xrdA 1xs0C 1xs3A 1xs9A 1xsfA 1xt5A 1xtjA 1xtuA 1xtxA 1xu1T 1xu6A 1xubA 1xv3A 1xvlB 1xw8A 1xwjA 1xwwA 1xxcA 1xxdD 1xyiA 1xzoA 1xzzA 1y0eA 1y0hB 1y0jB 1y0nA 1y0uA 1y12A 1y19B 1y43B 1y55X 1y5hB 1y5oA 1y5yA 1y66A 1y6dA 1y6qA 1y6uA 1y6xA 1y7mA 1y7pA 1y7uA 1y7xA 1y8mA 1y93A 1y96B 1y9bB 1y9xA 1ya5T 1yb0A 1ybiA 1ybjA 1ybkA 1ybuA 1yc5A 1yc61 1yd0A 1ydlA 1ye5B 1yfqA 1yfxA 1ygoA 1yhdA 1yhpA 1yi9A 1yk4A 1ykaA 1ykhA 1ykhB 1ylnA 1ymfA 1ymtA 1yn5A 1yn9A 1ynmA 1ynxA 1yo3A 1yo4A 1ypqB 1ypyA 1yqeA 1yqhA 1yqsA 1yr0A 1yr9A 1ys1X 1ys5A 1ytlC 1ytuB 1ytzT 1yua 1yudA 1yueA 1yug 1yujA 1yukA 1yukB 1yvcA 1ywhE 1ywkD 1ywmA 1ywsA 1ywuA 1ywyA 1ywzA 1yx0A 1yx6A 1yxeA 1yycA 1yyvA 1yzsA 1yzxA 1z00B 1z09A 1z0jB 1z1dB 1z1iA 1z27A 1z2fA 1z2gA 1z2mA 1z2nX 1z2uA 1z3rA 1z3xA 1z50A 1z65A 1z69A 1z6bB 1z6cA 1z70X 1z7aC 1z8mA 1z8rA 1z8sA 1z99A 1z9vA 1z9wA 1zaq 1zbe1 1zbfA 1zc4B 1zceA 1zd7A 1zdyA 1zeeA 1zfd 1zfo 1zfuA 1zg2A 1zgkA 1zglB 1zhcA 1zhvA 1zjiA 1zk4A 1zkeB 1zkkA 1zl0B 1zl8A 1zl8B 1zleB 1zlhB 1zljA 1zn5A 1zn6A 1zofB 1zoqA 1zoqC 1zoyC 1zp2A 1zpsA 1zpyG 1zr5B 1zrrA 1zs4D 1zshA 1zsoB 1zto 1ztpA 1ztsA 1zu1A 1zu2A 1zujA 1zv1A 1zw0B 1zw8A 1zwjB 1zwtA 1zxfA 1zxnA 1zyrE 1zz7A 1zzaA 2a0b 2a13A 2a19B 2a1vA 2a20A 2a26A 2a2kA 2a3kA 2a4hA 2a50A 2a5hB 2a5yB 2a6sB 2a6zA 2a7mA 2a7oA 2a7rB 2a7yA 2a8iA 2a93B 2a98A 2aamA 2ab5A 2ab9A 2absA 2acaA 2acmA 2acmB 2adzA 2ae6C 2aegA 2aehA 2afjA 2afwA 2agcA 2aghC 2ahmD 2aibA 2aivA 2aj7B 2ajfE 2ajhA 2akfA 2aliA 2am2A 2an1A 2anuE 2anxA 2apjD 2aqaA 2aqcA 2aqfA 2aqlA 2aqwA 2arcB 2arfA 2arhA 2asyA 2atrA 2avuE 2aw0 2aw2X 2awgA 2axlA 2axrA 2axtH 2axtI 2axtJ 2axtK 2axtL 2axtM 2axtU 2axvA 2axwA 2azeA 2azeC 2b1yA 2b34A 2b3nB 2b3wA 2b4aA 2b4eA 2b59A 2b68A 2b7eA 2b8mA 2b9dA 2ba2A 2baiA 2bayA 2bbg 2bdxA 2bemA 2beqA 2beqE 2bgpA 2bhuA 2bi6H 2bj1A 2bjfA 2bjnA 2bjyH 2bk9A 2bkdN 2bkvB 2bl8A 2blkB 2bm7A 2bmoA 2bmoB 2bn2A 2bn6A 2bngA 2bo5A 2bo9B 2bogX 2bopA 2boqA 2bpa2 2bpa3 2bpsA 2brfA 2bt8A 2bt9A 2btlA 2bukA 2bunA 2buoA 2bv9A 2bvbA 2bvmA 2bw4A 2bwqA 2bx9A 2byeA 2bz6L 2bzvA 2c0jA 2c0xA 2c1vA 2c24B 2c35B 2c3gA 2c3vB 2c4cA 2c5aB 2c5iT 2c5lC 2c5mC 2c5sA 2c5zA 2c6nB 2c71A 2c7hA 2c7pA 2c8sA 2c9gA 2calA 2caxD 2cc6A 2ccdA 2ccwA 2cdoC 2ce3E 2chhA 2conA 2cpmA 2cpo 2cprA 2cpyA 2cqoA 2cr8A 2crgA 2crqA 2cruA 2cs7A 2cskA 2csvA 2csyA 2cszA 2ct5A 2ctqA 2cw1A 2cweA 2cwqA 2cwsA 2cy7A 2cy9A 2cygA 2cz2A 2czsA 2d0pB 2d1pE 2d1sA 2d2mA 2d3dA 2d42A 2d46A 2d4hB 2d4qA 2drpA 2dynA 2eboA 2erjF 2erl 2ethA 2etxB 2eulA 2evrA 2ewcD 2ex3A 2ex4A 2ey4C 2ezi 2ezl 2ezvA 2ezwA 2f01B 2f06A 2f1vA 2f1xB 2f2bA 2f44A 2f46A 2f5gA 2f5tX 2f60K 2f69A 2f86B 2f8aA 2f8mB 2fa1A 2fa8B 2fckA 2fd4A 2fdmA 2fdn 2fdoA 2fe5A 2ffgA 2ffmA 2fftA 2fgdD 2fggA 2fgsA 2fhh2 2fhjA 2fi1A 2fj8A 2fk4A 2flgA 2fm1A 2fmr 2fnuB 2fomB 2fpeG 2fq3A 2fsqA 2fucA 2fy7A 2fz0A 2fz2B 2g03A 2gc9A 2gp8A 2hgf 2hntE 2hpaA 2ifo 2jdxA 2lefA 2lfb 2lisA 2ncm 2nef 2pfkA 2pni 2prf 2proB 2pth 2pvbA 2r63 2rlaA 2sak 2sivB 2sns 2tmvP 2tpsB 2vil 3caaB 3chbD 3cla 3csmA 3ezmA 3fua 3gsbA 3lzt 3ncmA 3pbh 3pviA 3seb 3vtk 3ygsP 3znbA 4aahA 4eugA 4hmgA 4mdhA 4mt2 4otaM 4sgbI 4ull 4vhbB 7a3hA 8abp 9apiB

**Table S2**. Test set proteins from PDBselect25, version Jan 2007. PDB code plus chain identifier, if present. There are a total of 559 chains.

1a6i 1a90 1acd 1afp 1ah9 1aj3 1avgI 1avqC 1ax3 1ay7B 1b3aA 1b8gA 1b9xC 1bc9 1bde 1brt 1bzbA 1bzg 1c53 1c5fI 1c94A 1c9kC 1cc5 1cif 1cjaA 1cjkA 1clh 1cr5A 1cxyA 1d4bA 1dcjA 1dec 1dfcA 1dg4A 1dkuA 1dowB 1dro 1dtjB 1du6A 1dv9A 1dy5A 1dz1A 1e1hA 1e29A 1e52A 1e5uI 1eazA 1ed7A 1edmB 1eioA 1eo2A 1eq2H 1exjA 1exkA 1f3mA 1f4iA 1f9yA 1fct 1fd9A 1ffkB 1fl0A 1fovA 1froA 1fsgA 1fu6A 1fw7A 1fyjA 1g1jA 1g2cK 1g2yA 1g3gA 1g47A 1g90A 1gc1G 1ggwA 1gh8A 1gjtA 1gks 1gnc 1gppA 1gsq 1gukB 1gur 1gxcA 1gxeA 1gy7C 1gzsB 1h31B 1h3lA 1h4gB 1h5pA 1h6fB 1h6vB 1h6xA 1h8bA 1hbkA 1hjqA 1hp3A 1hs7A 1hy9A 1hybA 1i1jB 1i21Y 1i24A 1i40A 1i69A 1ica 1ifc 1ifyA 1ig6A 1iieA 1ijqB 1iml 1isuA 1iu3C 1iwnA 1izmA 1j08A 1j0gA 1j22A 1j2jB 1j2lA 1j4wA 1jb0I 1je3A 1jekB 1jfmA 1jlxA 1jo0B 1jovA 1jr4A 1jrmA 1jt8A 1jvzA 1jzuA 1k1gA 1k1vA 1k3sB 1k4iA 1k61A 1k81A 1k8bA 1k8hA 1k8uA 1kbeA 1kftA 1kg0C 1kgsA 1kikA 1kj2B 1kngA 1kozA 1kp6A 1kqmB 1kqqA 1kt6A 1l0aA 1l1pA 1l5pA 1lb3A 1lbu 1lf7A 1lg7A 1lj2B 1lk9B 1lkxC 1lniA 1ltbC 1lu4A 1lucA 1m0iA 1m1sA 1m3cA 1m4jA 1m4uA 1m7tA 1m94A 1mjuH 1mjuL 1mk4A 1ml8A 1mm0A 1mn8B 1mnnA 1mw4A 1mxiA 1my7B 1mypA 1mzkA 1n0fA 1n2dC 1n32L 1n8bA 1nct 1nepA 1nglA 1nkd 1nkoA 1nn7A 1np6B 1nq4A 1nubA 1nziB 1o26C 1o4yA 1o5fL 1o7bT 1o7jA 1o82A 1o8xA 1oczM 1oewA 1oh0A 1ojlC 1oohA 1oqjA 1orgA 1ox9A 1oypA 1oz3A 1p19C 1p4oB 1p7aA 1p7mA 1paa 1pavA 1pd6A 1pd7A 1pffA 1pft 1plc 1ponB 1pq7A 1pv0A 1pvjA 1q02A 1q09A 1q2zA 1q3tA 1q6zA 1q7hA 1q7lB 1q80A 1q8gA 1q9fA 1qdnA 1qfqB 1qi1A 1qksA 1qmiA 1qmlA 1qstA 1qv1A 1qwdA 1qwyA 1qxyA 1qydA 1r1vB 1r26A 1r29A 1r2qA 1r48A 1r5rA 1r8gA 1rfbA 1rfeA 1rg8A 1rljA 1rqfH 1rqtA 1rw1A 1ryqA 1s2iA 1s31A 1s3cA 1s3zB 1s7aA 1s7oB 1s8kA 1s9aA 1se7A 1senA 1sfsA 1skhA 1smbA 1so0B 1sq9A 1sszA 1st9B 1su1C 1svfC 1sx5A 1t0bG 1t0vA 1t3yA 1t4wA 1t7dA 1t8kA 1tbaA 1te5A 1te7A 1teeA 1tg0A 1tig 1tit 1tizA 1tp6A 1tqjE 1tqzA 1trlA 1ttnA 1tujA 1tvgA 1tyhA 1u2fA 1u2hA 1u2nA 1u34A 1u4gA 1u57A 1u5dB 1ucrB 1ueyA 1ujlA 1ujrA 1uky 1ul7A 1un1B 1unqA 1upqA 1ursA 1uspA 1utg 1uw7A 1uwcA 1v1dA 1v1jA 1v4jA 1v5mA 1v61A 1v86A 1v88A 1v8fA 1v8oH 1v9wA 1vcuB 1verA 1vfyA 1vg5A 1vi3A 1vig 1vkhB 1vkkA 1vl7A 1vm9A 1vmhA 1vna 1vpjA 1vqo1 1vr7B 1vynA 1vziA 1w0nA 1w2lA 1w3bB 1w8xN 1wexA 1wf1A 1wf5A 1wg1A 1wg7A 1wggA 1wgsA 1wi3A 1wihA 1wijA 1wixA 1wjaA 1wkcA 1wkwA 1wl8A 1wlqF 1wlxA 1wlzB 1wqbA 1wrmA 1wrrA 1ww1B 1wwjA 1wx6A 1wx7A 1x1fA 1x2iA 1x2pA 1x31D 1x3vA 1x4lA 1x67A 1x6zA 1x8qA 1x9aA 1x9zA 1xc3A 1xd3C 1xdmZ 1xe6A 1xedC 1xg8A 1xhuB 1xiyA 1xk5A 1xlqA 1xm7A 1xmaA 1xmbA 1xo9A 1xswA 1xx1A 1xx6A 1xz8B 1y23B 1y2iA 1y30A 1y4mA 1y5eA 1y5nC 1y63A 1y7qA 1ybyB 1ycsB 1yd8G 1yelA 1yg9A 1ygmA 1yh5A 1yj8A 1yp5A 1ytfD 1yub 1yviB 1z0nA 1z0pA 1z0rA 1z0wA 1z1sA 1z3eA 1z3eB 1z4eA 1z5fA 1z67A 1z6oM 1z72B 1z9iA 1z9mB 1zdxA 1zeqX 1zk8B 1zkpB 1zl3A 1zmaA 1zndA 1zt3A 1zta 1zxjB 1zzkA 1zzpA 2a05A 2a07I 2a15A 2a2fX 2a41C 2a5gB 2a5zC 2a8kA 2a91A 2ad9A 2affA 2ahqA 2aj6A 2amiA 2aoaB 2asuB 2auaA 2aucC 2axtD 2axtE 2azvA 2b06A 2b43A 2b5yA 2b7fA 2b82A 2bds 2bf6A 2bf9A 2bfxC 2bhoA 2bkqC 2bp7B 2bugA 2bwfA 2c1mB 2c37A 2c5rA 2c74A 2carB 2cb6A 2ccmA 2cocA 2covG 2cpgC 2cpiA 2cq8A 2cqfA 2cqhA 2cqjA 2criA 2cswA 2ct0A 2ctmA 2cvbA 2cxaA 2czyA 2d7vB 2erbA 2errA 2esrA 2euiB 2ew0A 2eytA 2f1rA 2f4mB 2f62A 2f9jP 2fejA 2ffsB 2fg9A 2fiuA 2fk7A 2fk9A 2flsA 2fnoB 2fqmF 2frgP 2fvtA 2fxfB 2fyiC 2g8mA 2igd 2pil 2pleA 2rspA 2u1a 2u2fA 2vgh 3mspA 3vub 4cpaI 7i1b
